# Arthrodesis Using Pedicled Fibular Flap After Failed Infected Knee Arthroplasty

**Published:** 2011-11-21

**Authors:** Steve C. Minear, Gordon Lee, David Kahn, Stuart Goodman

**Affiliations:** ^a^Stanford University School of Medicine, Stanford, CA; ^b^Department of Surgery, University of California San Francisco, San Francisco, CA; ^c^Departments of Plastic and Reconstructive Surgery, Stanford University Medical Center, Stanford, CA; ^d^Department of Orthopaedic Surgery, Stanford University Medical Center, Stanford, CA

## Abstract

**Objective:** Severe bone loss associated with failed revision total knee arthroplasty is a challenging scenario. The pedicled fibular flap is a method to obtain vascularized bone for use in knee arthrodesis after failure of a total knee arthroplasty, with substantial loss of bone. **Methods:** We report 2 successful knee arthrodeses using this method in patients with infected, failed multiply revised total knee arthroplasties. The failed prosthesis was removed, and the bones were aligned and stabilized. The fibular flap was then harvested, fed through a subcutaneous tunnel, and placed within the medullary canal at the arthrodesis site. The soft tissue was closed over the grafts and flaps. **Results:** Two elderly women presented with pain and drainage from previous total knee arthroplasties after multiple revisions. Arthrodeses were performed as described, and both patients were pain-free with the knee fused at 1 year. **Conclusions:** Thus, pedicled vascularized flaps are a viable alternative in the treatment of failed revision arthroplasty with large segmental bone loss.

Total knee arthroplasty is a well-established procedure to treat arthritis refractory to conservative management.^1^ With the increasing number of these procedures performed,^2^ there is a resultant increase in the number of postoperative complications. Moreover, each revision surgery is progressively more difficult and associated with bone loss, which may eventually necessitate a salvage procedure.

Above-the-knee (AKA) amputation is a radical salvage, and many patients resist it. Knee arthrodesis is another option, typically performed using external fixation,^3^ intramedullary nailing,^3^^,^^4^ or plates and screws.^4^ These cases are often performed in the setting of severe bone loss, requiring extensive bone grafting.

Vascularized fibular grafts have been used to reconstruct defects of long bones in which segmental defects exist.^5^^-^8 Free fibular grafting requires advanced microsurgical techniques, and vascular complications such as arterial thrombosis or venous congestion may result.^9^^,^^10^ Pedicled fibular flaps have been used to obtain vascularized bone grafting during knee arthrodesis, but the complication rate can be as high as 92%.^11^^,^^12^

We report a knee arthrodesis procedure using a pedicled fibular flap technique, which has been successfully employed in 2 patients with infected, failed total knee replacements. This operation requires coordination between orthopedic and plastic surgeons and is a viable option in knee arthrodesis. This procedure has advantages over a standard arthrodesis: it can be performed in patients with severe bone loss because a large flap can be harvested. Also, the blood supply to the flap is intact and natural, which may lead to better healing and engraftment.

## MATERIALS AND METHODS: SURGICAL TECHNIQUE

An anterior approach to the knee joint was utilized. The hardware and cement were excised. The tissues and the femoral and tibial canals were debrided of devitalized tissue and retained cement. The tibial and femoral surfaces were recut to be flat and opposing. The femur and tibia were then stabilized with metallic fixation devices. The bone surfaces were apposed to minimize medial or lateral gapping.

The technique to harvest a fibula flap has been described previously.^13^^,^^14^ An incision was made on the lateral aspect of the leg over the fibula. An overlying skin paddle can be harvested with the fibula in situations where additional soft tissue is required. In the cases we present, a skin paddle was not required, and therefore, only the fibula was harvested. The peroneus muscle was retracted anteriorly, and a 1 mm cuff of muscle was left over the bone. Dissection proceeded anteriorly, and the anterolateral intermuscular septum and interosseous membrane were divided. The common peroneal nerve and associated superficial and deep branches, along with the anterior and posterior tibial vessels were identified and preserved. Osteotomies were performed distally and proximally, at the fibular neck and 6 cm above the ankle joint. The distal fibula was left for ankle joint stability. The tibialis posterior was divided and the vascular pedicle was divided distally. The flexor hallucis longus and other musculature of the deep posterior compartment were dissected from the bone, and subperiosteal dissection was made proximally to increase pedicle length. The prepared flap was then passed over the tibialis anterior and through a subcutaneous tunnel into the defect in the tibia and femur at the knee. The fibula was bivalved as necessary by a transverse osteotomy, creating 2 vascularized struts that each contained a full thickness of fibula and shared a periosteal blood supply. This construct was placed within the medullary canal after bone windows were created. The periosteum of the superficial bone strut was sutured to the surrounding soft tissues so it would not be displaced during closure. The pedicle was checked for signs of twisting of the vessels or compression from the bony tunnel. The patellar remnant and arthrotomy were closed over the grafts and flaps, and the wound was closed.

Branches of the vessels going to the posterior compartments required loupe magnification to dissect proximally to the tibioperoneal trunk. Topical papaverine was used for vasodilation and to prevent spasm. There were no intraoperative complications. Fixation included internal with/without external fixation or casting.

## RESULTS: CASE PRESENTATIONS AND OUTCOMES

Case 1. A 67 year-old woman had previously undergone a right-sided above-knee amputation postinfected multiply revised TKR and left-sided total knee arthroplasty. She was referred to our clinic in a wheelchair, 11 years postoperatively, for evaluation of left knee pain. Her left lower extremity functioned as a pivoting transfer leg. Two-staged revision surgeries had been performed: in the first surgery, an excision arthroplasty and placement of an antibiotic spacer were performed for a staphylococcal infection. After reimplantation, another operation was performed to revise the tibial bearing. Inflammatory markers remained elevated, with erythrocyte sedimentation rate and C-reactive protein peaking at 76 mm/h and 16.6 mg/L, respectively. The patient's pain was poorly controlled, and radiographs demonstrated loosening of the femoral and tibial components with severe bone loss. The decision was made to perform a staged arthrodesis of the knee to debride infected bone, reculture tissues, place an antibiotic spacer, and deliver appropriate intravenous antibiotics to clear the infection. In a second stage, an arthrodesis using a pedicled fibular flap was performed.

**Figure 1:**
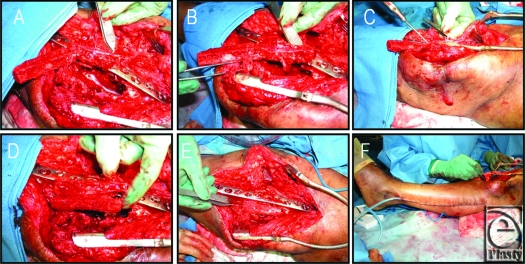
Intraoperative photographic documentation of case 1. (*a*) The pedicled fibular flap is bisected into a “double-barrel” configuration and fed through the subcutaneous tunnel. (*b*) The vascular pedicle is presented. (*c*) The flap is fitted into the arthrodesis site. (*d*) An additional free fibular graft is prepared to fit into the medial aspect of the arthrodesis site. (*e*) The flap and graft are fitted into the arthrodesis site. (*f*) The ipsilateral fibula harvest site is closed and the arthrodesis is completed.

**Figure 2:**
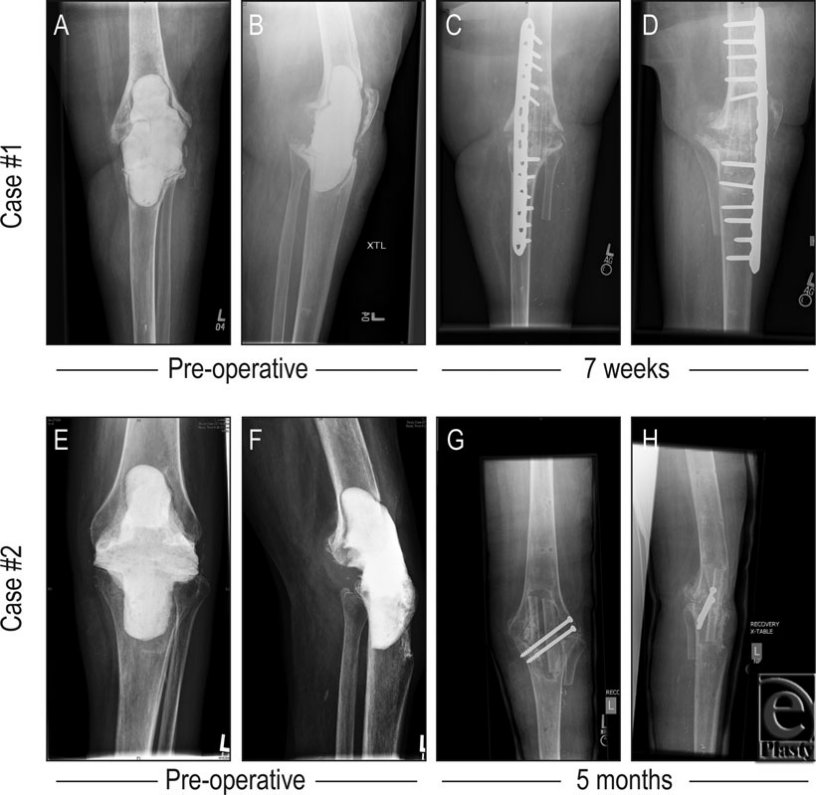
Radiographic documentation of cases. (*a*) Preoperative AP roentogram of infected knee joint of case 1. (*b*) Preoperative lateral roentogram of infected knee joint of case 1. (*c*) Postoperative AP. (*d*) lateral roentogram of case 1 at 7 weeks. (*e*) Preoperative AP roentogram of infected knee joint of case 2. (*f*) Preoperative lateral roentogram of infected knee joint of case 2. (*g*) Postoperative AP. (*h*) lateral roentogram of case 2 at 5 months.

The procedure detailed earlier was performed with minor variation. Attempts at apposing the femur and tibia resulted in a 6-cm gap anteriorly. These bones were fixed with an anteromedial plate, and a combination of cortical and locking screws were placed. Demineralized bone matrix (DBX, Synthes) was applied to the gap posteriorly. The fibular flap was prepared by dividing the bone into a double barrel strut with maintenance of the periosteal blood supply to each half. An additional 6-cm bone was harvested from the fibula as a free graft and used as an additional fitted strut into the medial aspect of the knee arthrodesis. See Figures 1 and 2 for photographic and radiographic documentation. The patient was seen 1 year after arthrodesis, with the knee solidly fused in position. At that point was mobilized to pivot and transfer to and from her wheelchair on her on one remaining leg.

Case 2. A 68-year-old woman who had undergone a left TKR 3 years ago was referred to our clinic because of persistent wound dehiscence and drainage. A revision had been performed, including patellectomy and extensor mechanism reconstruction. An irrigation debridement of the left knee was performed to excise necrotic bone fragments. Intraoperative cultures grew *S. viridians*, beta-streptococcus, and diphtheroids. Staged arthrodesis of the knee with pedicled fibular flap was planned.

The procedure detailed earlier was performed with minor variation. The exposed femur and tibia were apposed with good bony contact, achieved posteriorly with alignment of the knee into a slightly valgus and flexed position. Two lag screws were placed from lateral to medial, stabilizing the bones initially, and external fixation was placed anteromedially. Fixation was performed to allow placement of the fibular graft anteriorly. The harvested fibula flap was prepared as a single vascularized strut. Postoperatively, the external fixation was removed after 4 months and the limb casted. Vancomycin was given for 6 weeks intravenously. This patient was evaluated in clinic 12 months postoperatively, with her knee solidly fused. She was able to ambulate with minimal assistance without pain at her arthrodesis site. See Figure 2 for radiographic documentation of these cases.

## DISCUSSION

Bone grafts and osteoinductive materials are often needed in the treatment of failed knee arthroplasty to facilitate union of large gaps.^15^ Structural allograft usage may be complicated by nonunion, infection, and allograft fracture.^16^ In alloarthrodesis, nonunion rates are especially high relative to other graft types.^17^^,^^18^ Vascularized fibula flaps were developed as an alternative to the use of grafts. Pedicled fibular flap use is a technique that bypasses the need for microsurgical reconstruction as the blood supply is never interrupted.^19^^,^^20^ Its use is limited to the ipsilateral tibia and knee because it is tethered by the vascular bundle; thus, these flaps can be utilized around the donor site to achieve tibial defect reconstruction^19^ and as a salvage operation for failed knee arthroplasties with bone loss.

Slightly different fixation methods and flap utilization were employed in each case. In case 1, bone apposition of the host bone ends was marginal. An internal fixation bridging plate was placed anteromedially and bone graft was used to supplement the fixation. This was the best way to achieve good immediate stability and withstand the bending and torsional forces at the fusion site. In case 2, we were able to achieve good bone-to-bone apposition posteriorly, so we used interfragmentary compression screws posteriorly for internal fixation. We then used an external fixation device to supplement this method of fixation, as the screws alone were not sufficient to withstand the forces mentioned earlier, and plate fixation would not have allowed sufficient access to the defect for grafting.

In case 1, a “double barrel” strut configuration was used, while in case 2, the flap was not sectioned. Double strut utilization has an uninterrupted viable vascular supply across the periosteum^21^ and can be used to fill defects when a larger volume flap is required.^22^ Also in this case, an additional free fibular graft was available and was used to augment the construct. Therefore, the flap and fixation were tailored to the unique geometry of either defect.

The use of pedicled fibular flaps have recently been shown to have a promising role in the treatment of knee arthrodesis.^11^^,^^12^^,^^23^ However, microvascular techniques may be necessary if the pedicle is too short for proper graft impaction.^12^ At the time of this report, only a small percentage of our salvage procedures for failed knee arthroplasties utilize pedicled fibular flaps, because the defect can usually be addressed by limb shortening. In addition to bone stock, infection clearance, tissue viability, and ambulatory status of the patient, must be considered. Thus, fibular flaps are an option for salvage of the infected revision TKR with extensive bone loss.
